# Effects of decentralization on the functionality of health facility governing committees in lower and middle-income countries: a systematic literature review

**DOI:** 10.1080/16549716.2022.2074662

**Published:** 2022-08-12

**Authors:** Anosisye M Kesale, Christopher Mahonge, Mikidadi Muhanga

**Affiliations:** a Department of Local Government Management, School of Public Administration and Management, Mzumbe University, Morogoro, Tanzania; b Department of Policy Planning and Management, College of Social Sciences and Humanities, Sokoine University of Agriculture, Morogoro, Tanzania; c Department of Development Studies, College of Social Sciences and Humanities, Sokoine University of Agriculture, Morogoro, Tanzania

**Keywords:** Effects, health facility governing committees, functionality, lower and middle-Income countries, systematic literature review

## Abstract

**Background:**

Health facility governing committees (HFGCs) were established by lower and middle-income countries (LMICs) to facilitate community participation at the primary facility level to improve health system performance. However, empirical evidence on their effects under decentralization reform on the functionality of HFGCs is scant and inconclusive.

**Objective:**

This article reviews the effects of decentralization on the functionality of HFGCs in LMICs.

**Methods:**

A systematic literature review was conducted using various search engines to obtain a total number of 24 relevant articles from 14 countries published between 2000 and 2020. Inclusion criteria include studies must be on community health committees, carried out under decentralization, HFGCs operating at the individual facility, effects of HFGCs on health performance or health outcomes and peer-reviewed empirical studies conducted in LMICs.

**Results:**

The study has found varied functionality of HFGCs under a decentralization context. The study has found many HFGCs to have very low functionality, while a few HFGCs in other LMICs countries are performing very well. The context and decentralization type, members’ awareness of their roles, membership allowance and availability of resource to the facility in which HFGCs operate to produce the desired outcomes play a significant role in facilitating/limiting them to effectively carry out the devolved duties and responsibilities.

**Conclusion:**

Fiscal decentralization has largely been seen as important in making health committees more autonomous, even though it does not guarantee the performance of HFGCs.

## Background

The Alma Ata Declaration of 1978 identified community participation in health service delivery as a critical component of improving Primary Health Care (PHC). It is advocated for providing opportunities for health service users to directly participate in the design, implementation, and assessment of healthcare facility operations, to improve healthcare responsiveness, sustainability, and efficiency [1,2]. To incorporate communities in the planning, implementation, and evaluation of primary health care services, a variety of mechanisms have been developed by lower and middle-income countries (LMICs) [3]. The introduction or adoption of Health Facilities Governing Committees (HFGCs), also known as Community Health Committees, Village or Ward Health Committees, was one of the mechanisms utilized to improve community engagement in primary health care facilities [4]. These HFGCs, despite various terminologies used to name them in different countries, are community governance structures made up of community members who are responsible for representing the community in the planning, implementation, and management of health service delivery in primary health care facilities. Since the 1980s, the HFGCs have been working in various Health Sector Reforms (HSR) contexts, depending on the country’s distinctive path [5,6]. Some countries have combined community participation with decentralization measures, whereas others have not. Following the establishment of these HFGCs, the global health community has been eager to learn whether or not the existing HFGCs have achieved the desired health outcomes.

The decentralized health system is defined as the transfer of major decision-making powers and responsibilities for health services, such as planning, budgeting, and financial management from the central government or a large unit of local government to a smaller unit that is closer to the community [7–10]. Decentralization refers to a variety of measures, including de-concentration, in which authority and responsibility are transferred from the national level to regions or districts within the same ministry; Devolution, in which authorities and responsibilities are delegated to lower-level government structures; Delegation, in which semi-autonomous agencies are created to carry out functions that were previously controlled by the Ministry of Health; and Privatization, in which private owners assume responsibility and control [3,11]. Decentralization is adopted in the health sector to improve the performance of the health system, which improves the delivery of health services.

In the context of decentralization, it is widely accepted that community participation in primary health care facilities through various structures, such as HFGCs, can be functional enough in accomplishing their devolved functions and yield desired outcomes [11–13]. The goal of incorporating community involvement into primary health care was to increase citizen participation in the design, execution, and assessment of health service delivery in institutions such that the services generated reflected community preferences and needs. As a result, community members are expected to provide input during the management and governance of health facilities to make decisions that address community health concerns and promote community health, albeit this may not be the case in all health facilities. Indeed, under decentralized reform, HFGCs composed of community representatives elected or chosen by their community are likely to have a significant impact on health service delivery. This is because decentralization provides HFGCs with more options (functions and powers) and creates a conducive environment for them to carry out their duties [5,14]. As a result, the HFGCs are given crucial authority and decisions, such as revenue collection and expenditure, planning and administration of the health facility’s performance. The notion is that by forming HFGCs made up of community members and decentralized with additional functions and decision-making capabilities to govern health facilities, the community will be better served. HFGCs are better positioned and have more discretion than the central government to make new and more innovative judgments that are locally focused and maximize people’s preferences. Alma Ata’s dedication to establishing community engagement is congruent with the decentralization concept.

Empirical research, on the other hand, reveals that implementing decentralization in primary health care institutions and devolving authority to lower-level governance structures may not inevitably influence community engagement or HFGCs functionality. As Bossert and Abimbola [3,5] argue, agents devolved with discretionary powers and functions may choose not to exercise or take advantage of their devolved capabilities, continuing to behave and operate as they did before decentralization. As a result, certain agents, such as HFGCs, may not effectively carry out or be functional in accomplishing their devolved powers and duties to achieve the desired health objectives. As part of community participation in health care delivery, HFGCs would be expected to use devolved authorities to manage and govern primary health facility operations.

Despite the adoption of community participation in health service provision, empirical evidence on the functionality of HFGCs under decentralization as a part of community participation at the primary health care facility is lacking. The present empirical evidence is based on a small number of case studies or countries, which do not reflect the reality of the functionality of HFGCs in improving health outcomes in a decentralized setting. Three studies, for example, looked at the empirical evidence of the impact of decentralization on health outcomes [4,15,16]. Much of the research looked at the impacts of decentralization on health outcomes as well as the impact of accountability measures. The flaw in these empirical studies is that they did not look at the functionality or performance of HFGCs in a decentralized setting. Given the relevance of HFGCs in enhancing health system performance, a systematic review based on broader empirical research from lower and middle-income countries is required to assess the effect of decentralization on the functionality of HFGCs.

## Methods

A systematic literature review was conducted on empirical studies based on the protocols established by Cochrane Methods [17] and guided by the criteria articulated by PRISMA for systematic review reporting in the field of health [18,19]. The protocol and PRISMA are the following processes to be indicated: data search strategy, selection process, quality assessment, data extraction, result, data synthesis

## Data search strategy

We conducted a literature search from the different databases, such as PubMed, MEDLINE, JSTOR, Willey, Emerald Insight and Taylor & Francis to get empirical articles published from 2000 up to 2020. Articles published between 2000 and 2020 were selected because many lower and middle-income countries implemented decentralization in the 1990s, therefore by 2000 many countries were implementing it, and the impact of decentralization started to be realized. A manual search was also conducted on different web pages to get evaluation reports from different institutions. The selected databases were chosen because they publish public administration content; therefore, they adequately offered the needed articles for this study.

The goal of this study was to see how decentralization affected the functionality and efficacy of Health Facility Governing Committees in terms of enhancing health system outcomes. Because the amount to which powers are devolved to HFGCs varies by country under decentralization, this study looked at the functions of HFGCs in the context of the powers that have been devolved in the given country. These committees are responsible for guaranteeing the availability of critical medical equipment and pharmaceuticals, planning, budgeting, mobilizing and administering facility money, managing health personnel, and organizing communities to join community health funds, among other things. Since the term Health Facility Governing Committees is used differently in lower and middle-income countries, with some countries referring to them as health facility committees, community health committees, health user committees, and village or ward committees, this study searched for articles using similar terms in all terms amounting to community health committees. Words like ‘HFGC’, ‘Village health committees’ ‘community health committees’ ‘effectiveness,’ ‘functionality,’ ‘performance,’ ‘impacts,’ ‘outcomes,’ ‘effects,’ ‘outputs’ and ‘decentralization’ were paired with terms like ‘effectiveness of health facility committees’ or ‘performance of health facility governing committees’ to find articles.

## Selection process

All studies of various designs were eligible for the evaluation process if they met established inclusion and exclusion criteria. The following criteria were used to select eligible articles: the article had to be about health facility governing committees, (ii) it had to be original published articles or peer-reviewed articles, and (iii) it had to be conducted in lower and middle-income countries as defined by the World Bank [20], (iv) written in English language (v) the study’s goal was to determine the effectiveness, functionality, performance, or effects of the governing committee of a health facility on improving health outcomes. (vi) The factor of time (from 2000 to 2020). Papers that satisfied the aforementioned criteria were chosen and subjected to quality control and data selection.

## Quality assessment

To assess the quality of the selected studies, the researchers used a variety of assessment tools or criteria. The procedure for a systematic review of the literature was used for quantitative investigations [21] while for qualitative studies, the Critical Assessment Skills Program (CASP) was adopted https://casp-uk.b-cdn.net/wp-content/. The CASP indicators were utilized to choose which qualitative research should be included in the study, with 14 out of 29 qualitative studies matching the CASP requirements. The 14 studies chosen are those with a quality rating of more than 75% (high quality), of which 6 were chosen, and those with a rating of more than 50% but less than 75% (medium), of which 8 were chosen, and those with a rating of less than 50% (poor quality), of which 15 were not. These two assessment tools assisted in ensuring that the selected studies were methodologically appropriate for the investigation, that biases were avoided, and that their flaws were addressed. After the quantitative study assessment, the indicators ‘strong,’ ‘moderate,’ and ‘weak’ were utilized to represent the quality of the selected quantitative study.

## Data extraction

We retrieved information about the functionality or performance of HFGCs in carrying out their devolved powers and responsibilities at the facility level in the context of decentralization from each selected paper. The extraction was directed by the inclusion criteria set forth in order to successfully extract relevant information for the aim of this study. As a result, papers published before 2000 and after 2020 were eliminated, leaving just papers published between 2000 and 2020. We then looked at data from studies that looked at the functionality or performance of HFGCs in primary health care facilities exclusively (health centers, dispensaries, and health posts) that had been decentralized and given certain powers and responsibilities. This allowed information about certain HFGC’s responsibilities or tasks in a given facility to be extracted. We extracted information about the parameters influencing HFGC decentralized functionality or performance, as well as the health outcomes attained as a result of the HFGC functionality or performance, from each research.

## Results

### Included studies

**Figure 1.**
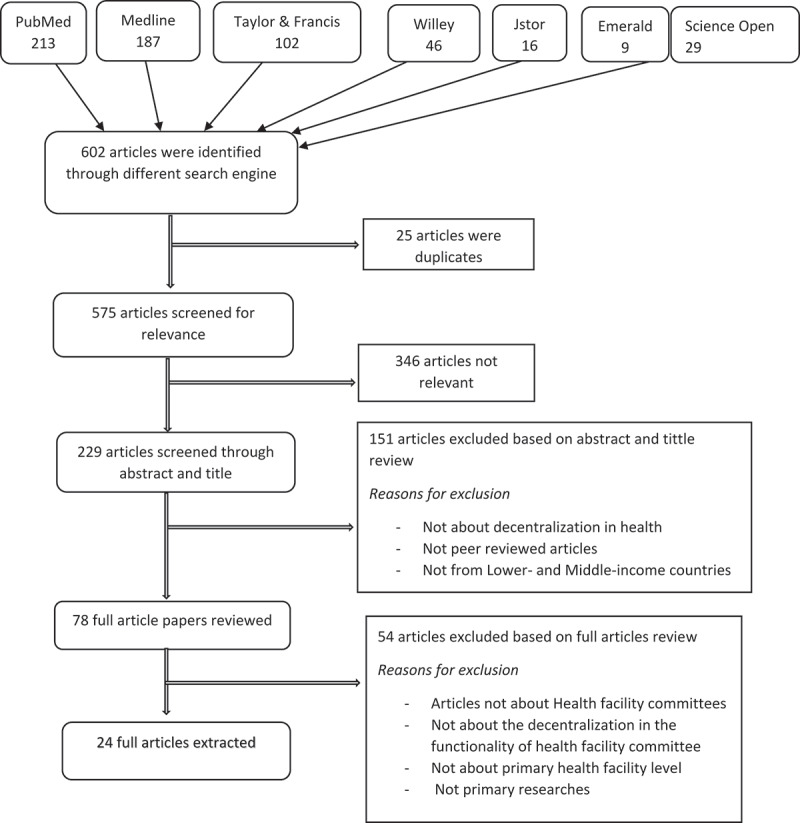
Literature search flow diagram.

The course of the literature review in this study is depicted in Figure 1. The method began with a total of 602 articles and titles being retrieved from various search engines, after which 25 articles were identified as duplicates from the 603 identified articles and titles, leaving the study with 575 articles and titles. Relevant articles and titles about decentralization in health care provision were evaluated for relevance; we ended up with 229 articles and titles following the screening. After reading the abstracts to evaluate if they were relevant to the study topic, 151 papers were eliminated, leaving 78. The reasons for the deletion are listed above. After a careful analysis, 24 articles qualified for extraction since they satisfied the predetermined criteria. Table 1 summarizes the articles qualified for extraction.

**Table 1.** Summary of the studies on the effectiveness and effects of Health Facility Governing Committees on Health system performance.

Author	Africa Region	Members of HFGC	Roles of HFGC	Functionality of HFGC	Factors affecting Functionality of HFGC	Health Outcomes
[23] Catherine Goodman., Antony Opwora., Margaret Kabare and Sassy Molyneux (2011) Health facility committees and facility management – exploring the nature and depth of their roles in Coast Province, Kenya	Kenya	**Committee members included the** • A health worker in charge as secretary • Between 8 and 18 community members. • The chair and the treasurer were chosen from the community members. • Most of the latter were farmers, though some were professionals such as teachers, and a few were community health workers	• oversee operations and management • To advise the community on matters • Articulate community interests • To facilitate a feedback process • To implement community decisions • Mobilize community resources • Raise funds Hire and fire subordinate staff	• Represent community • Oversee facility operations • Make final decision • Participate in outreach activities • Make final decision on the use of funds • An established good relationship with health workers • Participate in employing casual staff • Disciplining health workers	• Support from a higher level in training and resolving disputes • HFC allowance • introduction of fiscal decentralization through DFF • Availability of resources Negative • Lack of clarity of HFGC roles • less education	
[24] Waweru et al 2013 Are Health Facility Management Committees in Kenya are ready to implement financial management tasks: findings from a nationally representative survey	Kenya	- **Committee members** **included the** • A health worker in charge as secretary • Between 8 and 18 community members. • The chair and the treasurer were chosen from the community members. • Most of the latter were farmers, though some were professionals such as teachers, and a few were community health workers	• Supervise and control the administration of the funds allocated to the facilities; • Open and operate a bank account at a bank • Prepare work plans based on estimated expenditures; • Keep basic books of accounts and records of accounts of the income, expenditure, assets and liabilities of the facility • Prepare and submit certified periodic financial and performance reports • Keep a permanent record of all its deliberations.	• Determine how funds to be utilized • Raise issues, they have held in the community with facility staff • participate outreach activities • Sensitize the community on health matters • Raise funds • Participate in employing clerical staff • Participating in preparing annual facility plan	• A strong relationship between HFGC and worker • difference between municipal and non-municipal in controlling facility banks accounts • selection and representation of members **Negative** • education level • lack of awareness of their roles • allowances	• Mixed
[25] Karuga RN, Kok M, Mbindyo P, Hilverda F, Otiso L, Kavoo D, et al. (2019) “It’s like these CHCs don’t exist, are they featured anywhere?”: Social network analysis of community health committees in a rural and urban setting in Kenya	Kenya	• local leaders,• health facility staff and lay community members	• Provide leadership, • oversight in the delivery of community health services, • promote social accountability and mobilize resources for community health	• We’re not central actors in the exchange of health-related information. • Therefore, CHCs had little control over the flow of health-related information It emerged that CHCs were often left out in the flow of health-related information and decision-making, which led to demotivation	• Lack of information	
[26] Stephen Oswald Maluka1* and Godfrey Bukagile2 (2016) Community participation in the decentralized district health systems in Tanzania: why do some health committees perform better than others?	Tanzania		• Discuss and pass health center plans and budgets • Identify and solicit financial resources • Oversee the facility management • Ensure delivery of healthcare services • Link community with the health facility • Articulate community interest • Mobilize the community to join community health insurance	• perceived to be useful in sensitizing community members on CHFs, • supervised construction and rehabilitation of the health facilities, • managed health facility bank accounts and monitoring the provision of health services at the facility, including drugs and medical supplies.	• the financial incentive to the health facility committees • Managerial and leadership practices of the district health managers, including effective supervision and personal initiatives • Inadequate training and • low public awareness	
[27] Capurchande RD, Coene G, Roelens K, et al. Between compliance and resistance: exploring discourses on family planning in Community Health Committees in Mozambique.	Mozambique	• CHCs are composed of voluntary members, termed family planning facilitators, who are selected at the grassroots level.	• Mobilizing and counseling users/clients to use Family planning services	Inconsistence functionality of committees among facilities	• Training • sociocultural background • differences in knowledge as well • geographical • location	Not beneficial
[28] Emmanuel G. Kilewo & Gasto Frumence (2015) Factors that hinder community participation in developing and implementing comprehensive council health plans in Manyoni District, Tanzania Quality	Tanzania	• Community representatives • Health facility inchage • Private health services providers’ representatives • Faith-based health provider’s representatives • Village government representatives	• Participating in preparing health facility plan • role of facilitating the health facility Management Teams (HFMs) in planning and managing health initiatives in areas under them jurisdiction	Low participation of HFGCs in health Planning	• Low awareness of HFGC in participation in the planning process • Lack of financial resources allocated to support the implementation of HFGC activities • HFGC members lack management capacity • Lack of awareness of the roles and responsibilities of HFGC leads to poor participation in the development of CCHP Poor communication and information sharing• between CHMT and HFGC	
[29] Loewenson et al (2004) Assessing the impact of Health Centre Committees on health system performance	Zimbabwe		• facilitate people in the area to identify their priority health problems, • plan how to raise their own resources, • use information from the health information system and from communities in planning and evaluating their work • assess the impact of the health interventions in	• Drug availability • Sufficient number of staff• Increased resource placement	• Support from a higher level in training and resolving disputes • HFC allowance • introduction of fiscal decentralization through DFF • Availability of resources Negative • Lack of clarity of HFGC roles • less education	• Beneficial effects: Improved drugs availability, sufficient number of staff and improved allocation of finances
[30]Jean-Benoit Falisse a, L´eonard Ntakarutimana (2020) When information is not power: Community-elected health facility committees and health facility performance indicators	Burundi	• Members are elected by and from among the Health facility catchment population.	• Mobilization, management and allocation of the resources of the HF to ensure optimal implementation of the activities • check the integrity of the health infrastructure, drugs and equipment planning the development of HFs (quality of and access to services) and community health activities	• Failed to make major decisions to manage health facility	• Training to members • Information’s • Social-cultural factors • The context in which HFGC operated	• do not lead to visible improvements in terms of social• accountability, HF management, and use of and access to HF services.
[31]Elsbet Lodenstein 2017 Social accountability in primary health care in West and Central Africa: exploring the role of health facility committees	Benin, Guinea and Congo	• Composed of health workers and community members	• Monitoring of the budget formulation • execution, the management of user fees, • the establishment of drug inventories and orders. • promote financial transparency of pricing policies • Prevent extortion of patients and illegal drug sales. • disciplinary measures. HFCs are • contribute to conflict resolution between • the community and health providers	• They collect information about health challenges • Control and ensure availability of drugs prices • Manage facility finances • Manage performance of health workers • Provide feedback to the community • improved health worker presence, • the display of drug prices and replacement of poorly functioning health • workers.	• HFC leadership and synergy with other community structures	
[32] Shannon A. McMahon 2017 “We and the nurses are now working with one voice”: How community leaders and health committee members describe their role in Sierra Leone’s Ebola response	Sierra Leone’s			• They communicated Ebola-related messages to their peers, • enhanced provider understandings of community fears, • advocated for community needs within the health system. • Enabling mechanisms that supported community activities included the dual orientation of health committee members as community-members and • health system-affiliate	• Financial or in-kind • Recognition of the government’s limited human resource capacity to manage crises, • Recognition of the severity of Ebola, and • NGO supervision, -direction, and support Negative • inadequate supplies and resources, • criticism and distrust from their community, and • concerns or misunderstandings about the purpose of a task. • Contact tracers, in particular, highlighted that they were to receive weekly allowances but that this payment was irregular	• Positively contributed to combating Ebola
[33] Elsbet Lodenstein (2019) “We come as friends”: approaches to social accountability by health committees in Northern Malawi		• Composed of community representatives and facility staff	• bridging the communication gap between community and health staff, • inspection of facility conditions and drug stock, • formulating recommendations on facility equipment, • complaint management	• Monitored performance of health workers • Mediated Conflicts between health workers and patients • Reporting facility operations to the local authorities	• Committee capacities to judge health worker performance, • lack of clarity of roles and responsibilities • in upward and downward reporting processes	• Positive impacts on the performance of the facility staff
[34]Falisse, J. B., Meessen, J. Ndayishimiye, and M. Bossuyt, “Community participation and voice mechanisms under performance-based financing schemes in Burundi	Burundi			• Conflict with facility staff • Poor relationship with the community • was not able to monitor funds	• Members were not aware of their roles	
[35] Waweru et al (2016) Tracking implementation and (un)intended consequences: A process evaluation of an innovative peripheral health facility financing mechanism in Kenya	Kenya	• Community representatives		• Funds reach facilities on time • Funds are well monitored by the committee • Health workers are monitored by the committees	• Deepened decentralization	• Patient satisfaction improved • Proper and timely utilization of funds, Health workers are well monitored
[36]Daniel C. Ogbuabor & Obinna E. Onwujekwe (2018) Implementation of free maternal and child healthcare policies: assessment of the influence of context and institutional the capacity of health facilities in South-east Nigeria, Global Health Action	Nigeria			• HFCs are not involved in identifying eligible users of free care and managing free care refunds	• health facilities lacked service charters and complaint boxes • HFCs lack the legislative framework for the effective and efficient discharge of their functions	
[37]Olugbenga Oguntunde, Isa M. Surajo, Dauda Sulaiman Dauda, Abdulsamad Salihu, Salma Anas-Kolo and Irit Sinai5 (2018) Overcoming barriers to access and utilization of maternal, newborn and child health services in northern Nigeria: an evaluation of facility health committees	Nigeria	• one facility health provider and • 12–15 community residents. • Members represent all ethnic, religious, age, and gender groups who receive services in the facility. • Residents of hard-to-reach locales in the facility catchment area are • also included	• Find solutions to problems that people report about health facilities, as well as with • mobilizing the community to improve utilization of maternal and child health services, • sensitizing men and women in the community about the importance of obtaining maternal and child health services in the health facility.	• Mobilize community • Facilitate renovation of the facility • Provide linkage with communities and health workers • Ensured availability of medicine and medical equipment		• Facility health committees appear to have a positive influence on the quality of maternal and child health services • in the selected facilities
[38]Ngulube et al (2004) Governance, participatory mechanisms and structures in Zambia’s health system: An assessment of the impact of Health Centre Committees (HCCs) on equity in health and health care Centre for Health	Zambia		• Participate in planning and budgeting • Monitor expenditure • Mobilize communities to participate in health matters • Discussing issues relating to the health of the population	• Engaged in planning and budgeting • Monitored expenditure and revenue collection • Sensitizing community on health		Beneficial effects • Improved quality of service provision, improvement in monitoring of facility funds
[39]Mabuchi et al (2017) Pathways to high and low performance: factors differentiating primary care facilities under performance-based financing in Nigeria	Nigeria			• Better equipped facilities • Motivated staff • A good relationship with the community	• Contextual factors (competition and access) • Community engagement and support Performance and staff management	Beneficial effects: • Facilities are better equipped, and good management of health workers
[40]Gagan Gurung1, Sarah Derrett2, Philip C. Hill3 and Robin Gauld Nepal’s Health Facility Operation and Management Committees: exploring community participation and influence in the Dang district’s primary care clinics	Nepal	• clinic manager, • the village development committee chairperson, • elected members including school teachers, • female community health volunteers, • at least one of each of the followings: Dalit (a marginalized caste), Janajati (an ethnic group), and • female representatives (Gurung)	• To manage funds, human resources, and health programs, based on the principle of health sector decentralization • Infrastructure Local resources • Management of local staff • Management of permanent staff • Financial management • Health needs assessment	The depth of participation seems low • HFMC members did not consult with the community in a regular or systematic way, • There was no practice of providing feedback to the community.	• no democratic selection processes • HFMCs were influenced and captured by powerful elites.	
[41]Kamble RU, Garg BS, Raut AV, Bharambe MS. (2018) Assessment of functioning of village health nutrition and sanitation committees in a District in Maharashtra. Indian J Community Med	India	• Community health workers (called Accredited Social Health Activists (ASHAs)), • village nutrition and child development workers, • Auxiliary Nurse Midwives (ANMs), • Members of the locally elected government (called the gram panchayat), and • interested citizens.	• Conduct local health planning, and monitor the Anganwadi system and government health services. • Utilize the received facility funds • Preparation of village health plan • Preparation of village health register • organization of meetings and various health-related activities like health • camps, household survey, cleaning	• Low performing their duties and responsibilities • But at least Participate in preparing facility plan • Approved fund utilization Organized sensitization program	• Lack of Committee meetings with full attendance	little success in improving local health, sanitation, or nutrition
[42]Scott K, George AS, Harvey SA, Mondal S, Patel G, Ved R, et al. (2017) Beyond form and functioning: Understanding how contextual factors influence village health committees in northern India	India	• Community health workers (called Accredited Social Health Activists (ASHAs)), • village nutrition and child development workers, • Auxiliary Nurse Midwives (ANMs), • Members of the locally elected government (called the gram panchayat), and • interested citizens.	• Conduct local health planning, and monitor the Anganwadi system and government health services. • Utilize the received facility funds • Preparation of village health plan • Preparation of village health register • organization of meetings and various health-related activities like health • camps, household survey, cleaning	most • held monthly meetings, • identified a wide range of issues that required improvement, sought to address them largely by appealing to government officials	• Ingrained but negotiated social hierarchies; • Demoralizing resource and capacity deficits in government services undermining VHSNC legitimacy; • Contested VHSNC intersectoral authority despite widespread intersectoral needs and responsibility; • Fragmented and opaque accountability for supporting the VHSNC; • Underpinning power politics; and Parallel systems.	• little success in improving local health, sanitation or nutrition
[43]Rajpal Singh (2012) Limitations in the functioning of Village Health and Sanitation Committees in a North Western State in India	India	• Community health workers (called Accredited Social Health Activists (ASHAs)), • village nutrition and child development workers, • Auxiliary Nurse Midwives (ANMs), • Members of the locally elected government (called the gram panchayat), and interested citizens.	• Conduct local health planning, and monitor the Anganwadi system and government health services. • Utilize the received facility funds • Preparation of village health plan • Preparation of village health register • organization of meetings and various health-related activities like health camps, household survey, cleaning	• Failed to accomplish their duties such as • Raising awareness • Participating in planning Approving expenditure	• gaps in composition, formation and • The problems relating to the selection of members, • their training, • supportive supervision, • proper reporting and • responsive feedback mechanism	• little success in improving local health, sanitation, or nutrition
[44]Shirin Madon & S. Krishna (2017): Challenges of accountability in resource-poor contexts: lessons about invited spaces from Karnataka’s village health committees	India	• Community health workers (called Accredited Social Health Activists (ASHAs)), • village nutrition and child development workers, • Auxiliary Nurse Midwives (ANMs), • Members of the locally elected government (called the gram panchayat), and interested citizens.	• Conduct local health planning, and monitor the Anganwadi system and government health services. • Utilize the received facility funds • Preparation of village health plan • Preparation of village health register • organization of meetings and various health-related activities like health camps, household survey, cleaning	• mobilize community enrollment in the facility • Raising health awareness • Deciding on how to use funds Planning and monitoring village health		Moderately improved health service delivery
[45]Aradhana Srivastava1 2016 Are village health sanitation and nutrition committees fulfilling their roles for decentralized health planning and action? Mixed methods study from rural eastern India	India	• Community health workers (called Accredited Social Health Activists (ASHAs)), • village nutrition and child development workers, • Auxiliary Nurse Midwives (ANMs), • Members of the locally elected government (called the gram panchayat), and interested citizens	• Maintain data on the nutritional status of women and children, • Refer severely malnourished children to rehabilitation centers, • Prepare the nutritional components of the village health plan, and • Educate community members on nutritional issues. • Supervise Anganwadi Centres (AWCs), which are village-level nutrition and pre-school education centers, • Monitor the Village Health and Nutrition Day (VHND)	• Committees perform few of their specified functions for decentralized planning and action-conducting health awareness activities, • Supporting medical treatment for ill or malnourished children and pregnant mothers. Monitored drug availability with community health workers.	• irregular meetings, • members’ limited understanding of their roles and responsibilities, • restrictions on planning and fund utilization, and • weak linkages with the broader health system.	
Author	South America Region	Members of HFGC	Roles of HFGC	Functionality of HFGC	Factors affecting Functionality of HFGC	Health Outcomes
[46]Iwami and Petchey (2007) A CLAS act? Community-based organizations, health service decentralization and primary care development in Peru	Peru			• Committees were able to make major decisions such as the utilization of funds and • linking community with a facility		the improved user of satisfaction

### Data synthesis

The studies were divided into four categories: the first dealt with the membership of HFGCs in primary health care institutions, the second with the roles devolved to the HFGC as a result of decentralization, and the third with the roles devolved to the HFGC as a result of decentralization. The third category dealt with HFGC functionality in a decentralized environment, the fourth with the factors that influenced HFGC functionality, and the final category dealt with the effects of HFGC functionality on health service delivery. A meta-analysis was not performed in this investigation due to a number of limitations, including the research designs employed and the outcome measurement criteria used in each study. The quality assessment tool was adopted to ascertain the validity of the reviewed empirical studies since the instrument is recommended for covering empirical studies used in international development settings [22]

## The composition of health facility governance committees under decentralization

Many HFGCs were discovered to be made up of community representatives, reflecting community participation in the management and administration of health service delivery in many decentralized limits. The importance of community participation is mirrored in the composition of HFGCs, with community representatives accounting for the majority of HFGCs in the research examined. The following research, for example, has highlighted community representatives in HFGCs [23,24,27,28,30,32,37,41,42,44,47]. Health facility in charges or health facility staff have also been mentioned to be a member of the HFGCs who in many committees become HFGC secretaries [26,28,32,37,38,42]. Village governments or members of the local government in some countries are included in the HFGCs, as has been highlighted by the governing guidelines [26,28,40,42,45]. Gender representation has not been left out in the composition of HFGCs in many countries, this is evidenced by the special requirement of gender representation among community representatives in the HFGCs [26,28,40,45,48].

## The roles and powers of health facility governing committees under decentralization

The HFGCs were found to have been devolved with many responsibilities and roles to fulfill in the course of governing and administering primary health facilities, according to the extracts investigations. Some of the responsibilities include managing and overseeing facility operations [23,25,26], to articulate community interest and address community health matters [23,26,30,33,37,42], participating in planning and budgeting [23,26,31,38,42,45,49,50]. Other common functions of HFGCs are to mobilize facility resources such as funds and other materials [23,25,26,33,37,38], managing the performance of health workers, including hiring and firing [23,26,40] and facilitate feedback to community and health facilities [23,26,31,33,38].

## The functionality of health facility governing committees in the decentralized health system

The extracts reviewed have highlighted the functionality of HFGCs as a means of facilitating community participation in the decentralized context. The results indicate that the functionality of HFGCs in many countries is still very low and below the expectation of pioneers of health reforms even though in other countries HFGCs are functioning well. Some studies that have shown that HFGC functionality under the decentralization context is very limited [25,27,28,30,34,36,40,41,43,45]. On the other hand, other studies have found that HFGCs are functioning very well and accomplishing their duties and responsibilities to a large extent [23,24,26,29,31–33,37,38,42,44,46].

Indeed, the studies have highlighted some of the roles which are performed well by the majority of the HFGC such as engagement in the planning and budgeting process [23,24,26,35,38,46]. Monitored performance of health workers [31–33,37], finding solutions to the community health problems [26,32,37,42]. Other HFGCs were doing well in mobilizing and sensitizing communities on health programs [24,26,42]. Meanwhile, HFGC has been found to be ineffective in other circumstances, failing to engage in budgeting and planning, linking community and health facilities, convening HFGC meetings, and making other significant decisions that could improve health service delivery [25,30,34,40,41].

## Factors influencing the functionality of health facility governing committees under decentralization

A variety of factors have been linked to the functionality of the HFGCs in developing nations’ decentralized health systems. These variables are linked to both positive and bad functionality in various ways. The highlighted factors found to be associated with HFGCs functionality are HFGC membership allowance [23,24,26,32], awareness on the HFGC roles and powers [23,29,30,33,34], introduction of fiscal decentralization (23,24,28). Other factors are Training to committees [27,30,43], availability of resources [23,24,26,28,42], context in which the facility operates [30,32,36,43,45]. Furthermore, social norms, leadership, HFGC selection and composition, and even the ways of recruiting members were found to be linked to HFGC functionality.

## Discussion

In lower- and middle-income countries, expanding decentralization in primary health care institutions is proposed as a foundation for improving community engagement in the management and control of health service delivery. Indeed, decentralization is claimed to give community health structures like HFGCs considerable powers and functions, empowering them and increasing the depth of engagement in boosting health service delivery at the facility level. This is in line with the Alma Ata Declaration’s aim of community participation in the design, implementation, and administration of their health care. Therefore, it is expected that decentralization would positively influence the functionality of HFGCs and be able to deliver their mandates. To determine the functionality of HFGCs in the decentralized health system in primary health care, we conducted a systematic literature review. Twenty-four studies were reviewed from 13 countries in 3 regions. Linked matters relating to the functionality of HFGCs were assessed including the roles of HFGCs, the membership of the HFGCs, the functionality of HFGCs, the factors influencing the functionality of HFGCs and the effects of HFGCs on health outcomes under decentralization. In a decentralized setting, we discovered functionality inconsistency among HFGCs in lower- and middle-income countries, with the majority of HFGCs having limited functionality even after decentralization.

The study discovered that HFGCs in many nations from various locations, such as Asia, Africa, and South America, had similar compositions, with community representatives and other government staff in all of them. These committees have found that the bulk of their members come from communities with few government officials, such as village or local government representatives, and facility employee representatives, to reflect the community. This means that the HFGC was created to increase community participation in defining health service delivery and to be responsive to community needs and preferences. The survey also discovered that the majority of HFGC roles are similar across countries and locations. In many countries, the role of the HFGC is to connect communities with health facilities, participate in planning and budgeting, approve facility expenditure, mobilize and sensitize communities about various health programs, and manage health worker performance, including hiring and firing some clerical staff. Other responsibilities include directing facility administration, managing health facility finances, discussing and addressing community health concerns, and managing health facility finances.

Decentralization of powers and functions to HFGCs at the primary health care facility level does not guarantee effective HFGC functioning or increased community participation at the facility level, according to the study’s findings. This is because, in many nations, HFGCs have been shown to have a variety of performances in their decentralized roles. After decentralization, it was envisaged that HFGC would be able to carry out its tasks and responsibilities more effectively and have an impact on health service delivery. However, when it comes to reality, the results show that this is not the case. This is in line with Bossert’s [5] belief that giving grassroots organizations more decision-making power does not guarantee that change will occur. Many HFGCs have discovered that various circumstances are preventing them from realizing their full potential. For example, HFGCs fail to fulfill their responsibilities because they are unaware of the scope of their responsibilities and powers, while others are working with insufficient resources, lack of support from higher levels, and small committee composition. Allowance to members of the HFGCs has been found to be critical in encouraging them to carry out their responsibilities, even though members declare that they are working freely for the benefit of the community.

The ‘allocative efficiency’ principle states that because of the information advantage that sub-national institutions or facility-level institutions have over the national government, they can improve health outcomes through proper resource allocation. However, the situation in some health facility committees is a little different. These committees have been proven to have fewer effects on the performance of the health system than expected, some of which contradict the allocative efficiency thesis. The performance variances or efficacy of health facility committees in low- and middle-income nations can be explained by a variety of factors. The decentralization environment that different lower and middle-income nations have experienced or implemented is one key influence.

## Conclusion

Decentralization of the health system promised the empowerment of subnational health institutions, which would be particularly effective in carrying out their tasks in enhancing primary health care outcomes. However, reality differs from assumptions, as many studies have shown that decentralization alone cannot improve health service delivery at the primary health care facility level by influencing community engagement and the functionality of community governance structures, such as HFGCs. Instead, the setting in which HFGCs function, as well as the adoption context for decentralization, is critical to achieving the benefits of HFGCs and decentralization in general.

## Paper context

Governing committees for health facilities were formed to encourage community participation in the delivery of health services. Decentralization is thought to have a significant impact on the HFGCs’ ability to govern service delivery. The findings reveal that HFGCs have promising effects, despite the fact that they are still far from fulfilling their full potential. These committees should be capacitated in their tasks and powers and given full autonomy in order to realize their full potential.
